# The Relative Importance of Cytotoxins Produced by Methicillin-Resistant *Staphylococcus aureus* Strain USA300 for Causing Human PMN Destruction

**DOI:** 10.3390/microorganisms12091782

**Published:** 2024-08-28

**Authors:** Tyler K. Nygaard, Timothy R. Borgogna, Kyler B. Pallister, Maria Predtechenskaya, Owen S. Burroughs, Annika Gao, Evan G. Lubick, Jovanka M. Voyich

**Affiliations:** Department of Microbiology Cell Biology, Montana State University, Bozeman, MT 59718, USA; timothy.borgogna@montana.edu (T.R.B.); mpredte@gmail.com (M.P.); owen.burroughs@vanderbilt.edu (O.S.B.); annikagao3@gmail.com (A.G.); evlubick@gmail.com (E.G.L.); jovanka@montana.edu (J.M.V.)

**Keywords:** *Staphylococcus aureus*, MRSA, USA300, neutrophil, leukocidin, cytotoxicity, virulence, pore-forming toxin, PVL, LukGH

## Abstract

*Staphylococcus aureus* (*S. aureus*) is a prominent Gram-positive bacterial pathogen that expresses numerous cytotoxins known to target human polymorphonuclear leukocytes (PMNs or neutrophils). These include leukocidin G/H (LukGH, also known as LukAB), the Panton–Valentine leukocidin (PVL), γ-hemolysin A/B (HlgAB), γ-hemolysin B/C (HlgBC), leukocidin E/D (LukED), α-hemolysin (Hla), and the phenol-soluble modulin-α peptides (PSMα). However, the relative contribution of each of these cytotoxins in causing human PMN lysis is not clear. In this study, we used a library of cytotoxin deletion mutants in the clinically relevant methicillin-resistant *S. aureus* (MRSA) isolate LAC (strain ST8:USA300) to determine the relative importance of each for causing human PMN lysis upon exposure to extracellular components as well as following phagocytosis. Using flow cytometry to examine plasma membrane permeability and assays quantifying lactose dehydrogenase release, we found that PVL was the dominant extracellular factor causing human PMN lysis produced by USA300. In contrast, LukGH was the most important cytotoxin causing human PMN lysis immediately following phagocytosis with contributions from the other bicomponent leukocidins only observed at later time points. These results not only clarify the relative importance of different USA300 cytotoxins for causing human PMN destruction but also demonstrate how two apparently redundant virulence factors play distinctive roles in promoting *S. aureus* pathogenesis.

## 1. Introduction

*Staphylococcus aureus* is a common Gram-positive bacterium that is a major cause of human morbidity and mortality, associated with more than 1.1 million deaths worldwide [1] and responsible for over 1.7 billion US dollars in medical costs in the United States alone in 2019 [2,3]. Widespread antibiotic resistance and the lack of an effective vaccine limit our ability to treat and prevent infections caused by this pathogen. In particular, methicillin-resistant *S. aureus* (MRSA) identified by pulsed-field gel electrophoresis (PFGE) as USA300 is currently the dominant clinical isolate in the United States [4,5,6,7,8,9].

The capacity of *S. aureus* to cause a wide variety of disease in both humans and animals is attributed to the diverse and seemingly redundant array of virulence genes expressed by this organism [10,11]. These include numerous adhesins that bind to specific host molecules, pore-forming toxins that impair the integrity and function of different host cells, and immunomodulatory proteins that directly manipulate the host immune response. For example, the USA300 genome encodes more than seven prominent pore-forming toxins known to be active against different human cell types [12,13,14,15,16,17,18]. These include five bicomponent leukocidins - leukocidin G/H (LukGH, also known as LukAB), Panton–Valentine leukocidin (PVL), γ-hemolysin A/B (HlgAB), γ-hemolysin B/C (HlgBC), and leukocidin E/D (LukED) - as well as α-hemolysin (Hla) and the phenol-soluble modulin-α peptides (PSMα). Given the multitude of *S. aureus* virulence genes with apparent overlapping function, parsing out their relative importance in promoting different aspects of disease has remained difficult.

Polymorphonuclear leukocytes (PMNs or neutrophils) are the most common circulating immune cells in humans and play an important role curtailing *S. aureus* pathogenesis [19,20,21]. Previous studies have shown that human PMNs are susceptible to intoxication by HlgAB [22,23,24,25,26,27,28,29,30], HlgCB [22,23,24,25,26,27,29,30,31,32,33], PVL [22,23,24,29,30,31,32,33,34,35,36,37,38], LukGH [22,24,28,29,30,35,39,40,41,42,43,44,45], LukED [22,23,24,30,46], Hla [39,47], and PSMα [37,39,48,49,50,51,52,53]. However, the majority of this research has examined the cytotoxicity of single virulence factors and often relied upon purified proteins used in excess to what is normally produced by *S. aureus*. An unbiased comprehensive analysis comparing relevant concentrations of each of these virulence factors has been lacking. As such, the contribution of each pore-forming toxin produced by *S. aureus* towards lysing human PMNs is not clear. 

In this study, we used a library of cytotoxin deletion mutants in USA300 to determine the relative importance of each in causing human PMN destruction. Our results show that PVL is the dominant extracellular cytotoxic factor causing PMN lysis that is produced by USA300, while LukGH is the primary cause of initial PMN destruction following phagocytosis of USA300. These findings show the potency of PVL, and LukGH largely depends upon the context of intoxication and indicate these bicomponent leukocidins play distinct roles in promoting pathogenesis.

## 2. Materials and Methods

### 2.1. Bacteria Strains and Culture Conditions

Bacteria were cultured at 250 rpm and 37 °C. Overnight cultures grown in tryptic soy broth (TSB; EMD Millipore, Burlington MA, USA) were used to start subcultures in a 14 mL culture tube containing 5 mL TSB (1:100 dilution) unless otherwise stated. *S. aureus* PFGE-type USA300 strain LAC used in this study has been described previously [54]. Genomic mutations of mutants used in this study (Table 1) were performed as previously described [55,56,57,58,59] using primers listed in Table 2. All mutants used in this study underwent whole-genome sequencing and the breseq computational pipeline [60] to confirm the desired mutation as well as verify that no off-target mutations have occurred or that these strains have lost endogenous plasmids. To generate complementary plasmids, PCR amplification was performed using primers listed in Table 2 with the indicated restriction enzyme sites and cloned into pRB473 as previously described [61].

### 2.2. Human PMN Purification

Human polymorphonuclear leukocytes were isolated under endotoxin-free conditions (<25.0 pg/mL) using freshly drawn heparinized venous blood from healthy donors with written informed consent as previously described [56,57,58,61,62,63,64,65,66]. Cell viability and purity of preparations were assessed using a FACSCalibur (BD Biosciences, Franklin Lakes, NJ, USA) or SE520EON flow cytometer (Stratedigm, San Jose, CA, USA) to ensure that only preparations containing ≥95% PMNs with ≥95% viability were used. Human PMNs were used immediately following isolation.

### 2.3. Cytotoxicity Assays

Intoxication of PMNs with extracellular *S. aureus* proteins was performed as previously described [56,57,58,61,63,65,66]. Briefly, *S. aureus* strains subcultured for five hours in TSB were centrifuged (5000× *g* for 5 min), and the collected supernatant was immediately tested for PMN cytotoxicity. To examine the cytotoxicity of *S. aureus* supernatants grown in different media types, *S. aureus* was subcultured in Luria–Bertani broth (LB), Todd–Hewitt broth with 0.2% yeast extract (THY), or brain–heart infusion broth (BHI) where indicated. To intoxicate PMNs, 20 µL of freshly collected *S. aureus* supernatant was combined with 100 µL Roswell Park Memorial Institute (RPMI) 1640 Medium (Corning Cellgro, Corning, NY, USA) containing 5 × 10^5^ freshly purified human PMNs in a serum-coated well of a 96-well plate. Intoxicated PMNs were incubated at 37 °C for 60 min, or other times where indicated, and then examined for plasma membrane permeability to propidium iodide (PI; ThermoFisher Scientific, Waltham, MA, USA) using a FACSCalibur (BD Biosciences) or SE520EON (Stratedigm) flow cytometer. Lactate dehydrogenase (LDH) release was measured using a Cytotoxicity Detection KitPLUS (Roche Diagnostics, Indianapolis, IN, USA) with an Epoch2 microplate spectrometer (BioTek Instruments, Winooski, VT, USA) following the manufacturer’s protocol.

### 2.4. Phagocytosis Assays

An examination of human PMN plasma membrane permeability following phagocytosis of live *S. aureus* was performed as previously described [56,57,61,63,65,66]. Briefly, subcultured *S. aureus* was harvested at mid-exponential (ME) growth by centrifugation (5000× *g* for 5 min) and opsonized with 20% normal human serum for 15 min at 37 °C. Freshly cultured and opsonized bacteria were washed with DPBS and then 1 × 10^7^ colony-forming units (CFUs) in 100 µL of Dulbecco’s Phosphate-Buffered Saline (DPBS) was combined with 100 µL of RPMI containing 1 × 10^6^ freshly purified human PMNs in a serum-coated well of 96-well plate (10:1 ratio of bacteria to PMN). Phagocytosis was synchronized by centrifugation (500× *g* for 5 min at 4 °C) in an Allegra X-15R centrifuge (Beckman Coulter, Indianapolis, IN, USA) and samples were incubated at 37 °C for 90 min unless otherwise stated. Following incubation, human PMNs were analyzed for PI plasma membrane permeability and LDH release as described above.

## 3. Results

### 3.1. Bicomponent Leukocidins Are the Primary Extracellular Cytotoxic Component Produced by USA300 against Human PMNs

**Figure 1:**
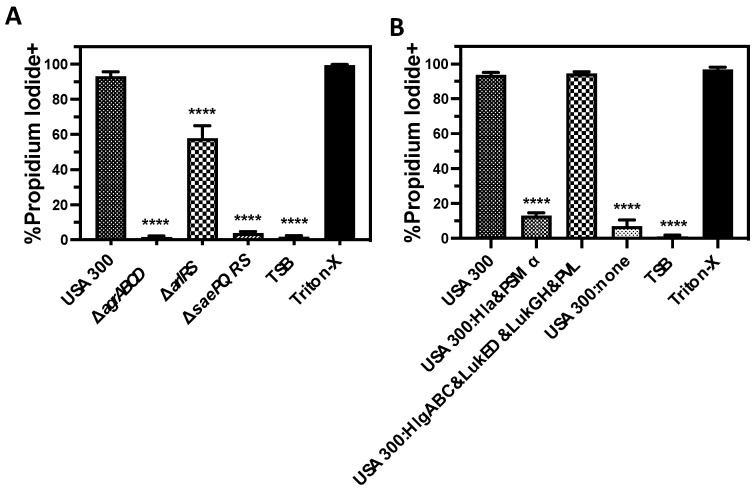
Human PMN plasma membrane permeability caused by extracellular factors produced by USA300 is primarily mediated by bicomponent leukocidins. Flow cytometry was used to assess the percentage of purified human PMNs permeable to propidium iodide after exposure to supernatants from (**A**) USA300, a deletion mutant of the AgrABCD two-component system in USA300 (USA300Δ*agrABCD*), a deletion mutant of the ArlRS two-component system in USA300 (USA300Δ*arlRS*), or a deletion mutant of the SaePQRS two-component system in USA300 (USA300Δ*saePQRS*) as well as (**B**) USA300, a deletion mutant of the bicomponent leukocidins *hlgABC*, *lukED*, *lukGH*, and *pvl* in USA300 but that still expresses Hla and PSMα (USA300:Hla&PSMα), a deletion mutant of the pore-forming toxins hla and psmα but that still expresses the bicomponent leukocidins (USA300:HlgABC&LukED&LukGH&PVL), or a deletion mutant of both the bicomponent leukocidins and pore-forming toxins in USA300 (USA300:none). For both panels, PMNs were also treated with tryptic soy broth (TSB) alone or TSB with 0.5% Triton X-100 (Triton-X). All data are the mean ± SEM of at least 3 independent experiments with **** *p* ≤ 0.0001 relative to USA300 as determined by repeated measures one-way ANOVA with Tukey’s multiple comparison test.

*S. aureus* produces numerous proteins reported to be cytotoxic against human PMNs. The SaePQRS and AgrABCD two-component systems are both known to regulate the production of most of these cytotoxic factors including the bicomponent leukocidins PVL, HlgAB, HlgCB, LukED, and LukGH as well as Hla [11,13,16,59,61]. AgrABCD also regulates additional exotoxins including PSM*α* [67]. In addition, the ArlRS two-component system has been shown to upregulate PVL, HlgCB, and LukGH [68]. To initially examine the relative importance of these different cytotoxins for causing human PMN destruction, we measured the plasma membrane permeability of primary human PMNs intoxicated with supernatants from wild-type USA300 or deletion mutants of USA300 in the SaePQRS (ΔsaePQRS), AgrABCD (ΔagrABCD), or ArlRS (ΔarlRS) two-component systems (Figure 1A). Congruent with previous findings [56,57], loss of the SaePQRS two-component system completely abrogated plasma membrane permeability caused by USA300 supernatants. As expected, the deletion of AgrABCD also eliminated the cytotoxicity of USA300 supernatants. We also noted a smaller but significant decrease in PMN plasma membrane permeability caused by USA300 extracellular factors when ArlRS was removed, similar to recently published findings by others [68]. 

To determine which class of cytotoxins is the most responsible for human PMN lysis, we examined the cytotoxicity of extracellular proteins produced by a USA300 mutant lacking all of in the bicomponent leukocidins but that still produces Hla and PSMα (USA300:Hla&PSMα), a USA300 mutant lacking Hla and PSMα but that still expresses HlgAB, HlgCB, PVL, LukGH, and LukED (USA300:HlgABC&LukGH&PVL&LukED), as well as USA300 that does not express Hla, PSMα, HlgAB, HlgCB, PVL, LukGH, or LukED (USA300:none). We found that PMN plasma membrane permeability following intoxication with extracellular proteins was primarily driven by the bicomponent leukocidins while no significant increase (*p* = 0.3696 relative to USA300:none) in plasma membrane permeability was observed with Hla and PSMα expression (Figure 1B). 

### 3.2. PVL Is the Prominent Cytotoxic Extracellular Factor Produced by USA300 That Causes Human PMN Destruction

**Figure 2:**
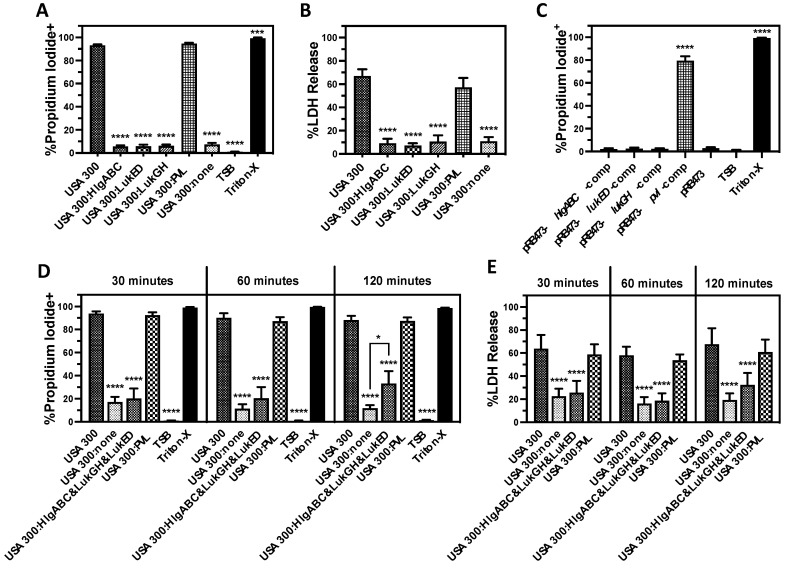
PVL is the major extracellular factor produced by USA300 causing human PMN lysis. Purified human PMNs intoxicated with supernatants from USA300, deletion mutants of multiple bicomponent leukocidins in USA300 but that still express HlgAB and HlgCB (USA300:HlgABC), LukED (USA300:LukED), LukGH (USA300:LukGH), PVL (USA300:PVL), or none of the bicomponent leukocidins (USA300:none) for 60 min were then assessed for (**A**) plasma membrane permeability to propidium iodide and (**B**) lactate dehydrogenase (LDH) release. (**C**) USA300 lacking all of the bicomponent leukocidins was transformed with the pRB473 control or pRB473 encoding *hlgABC*, *lukED*, *lukGH*, or *pvl* and supernatants from these strains examined for the ability to cause human PMN plasma membrane permeability. Purified human PMNs intoxicated with supernatants from USA300, a deletion mutant of all the bicomponent leukocidins in USA300 (USA300:none), a deletion mutant of *pvl* that still expresses the other bicomponent leukocidins (USA300:HlgABC&LukED&LukGH), or a deletion mutant of all the bicomponent leukocidins except for *pvl* (USA300:PVL) for 30, 60, or 120 min were assessed for (**D**) plasma membrane permeability to propidium iodide and (**E**) LDH release. For all panels, PMNs were also treated with tryptic soy broth (TSB) alone or TSB with 0.5% Triton X-100 (Triton-X). All data are the mean ± SEM of at least 3 independent experiments with * *p* ≤ 0.05, *** *p* ≤ 0.001, and **** *p* ≤ 0.0001 relative to USA300 (Panels (**A**,**B**,**D**,**E**) or pRB473 (panel (**C**)) as determined by repeated measures one-way ANOVA with Tukey’s multiple comparison test.

**Figure 3:**
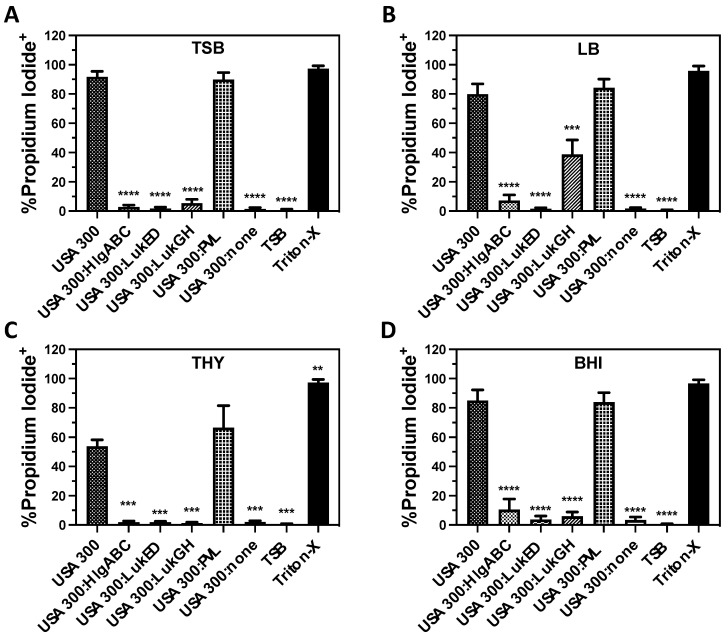
The influence of different culture media on PMN plasma membrane permeability caused by extracellular factor produced by USA300. Flow cytometry was used to assess the percentage of purified human PMNs permeable to propidium iodide after exposure to supernatants from USA300, deletion mutants of multiple bicomponent leukocidins in USA300 but that still express HlgAB and HlgCB (USA300:HlgABC), LukED (USA300:LukED), LukGH (USA300:LukGH), PVL (USA300:PVL), or none of the bicomponent leukocidins (USA300:none) that were subcultured in (**A**) tryptic soy broth (TSB), (**B**) Luria–Bertani broth (LB), (**C**) Todd–Hewitt broth with 0.2% yeast extract (THY), or (**D**) brain–heart infusion broth (BHI). All data are the mean ± SEM of 3 independent experiments with ** *p* ≤ 0.01, *** *p* ≤ 0.001, and **** *p* ≤ 0.0001 relative to each strain grown in TSB as determined by repeated measures one-way ANOVA with Tukey’s multiple comparison test.

To determine the contribution of each of the bicomponent leukocidins towards PMN lysis caused by extracellular factors produced by USA300, we examined USA300 deletion mutants that express only one bicomponent leukocidin (Figure 2). Human PMNs intoxicated with extracellular proteins produced by a USA300 deletion mutant of *hlgABC, lukGH*, and *lukED* but that still has *pvl* (USA300:PVL) exhibited plasma membrane permeability (Figure 2A) and lactate dehydrogenase (LDH) release (Figure 2B) equivalent to wild-type USA300. In contrast, the expression of any other single bicomponent leukocidin did not have an observable influence on PMN destruction caused by USA300 supernatants. Only after intoxication for 120 min could a modest increase in plasma membrane permeability and LDH release be detected in PMNs exposed to supernatants from USA300 expressing HlgAB, HlgCB, LukGH, and LukED, while the expression of PVL alone caused PMN lysis equivalent to the USA300 wild-type at all the times examined (Figure 2A,B). In addition, only the complementation of USA300 lacking all bicomponent leukocidins with a plasmid encoding *pvl* rescued the cytotoxicity of extracellular proteins against human PMNs while the reintroduction of the other bicomponent leukocidins had no observable influence on cytotoxicity (Figure 2C). PVL remained the dominant extracellular cytotoxic factor causing PMN lysis during growth in tryptic soy broth (TSB), Luria–Bertani broth (LB), Todd–Hewitt broth with yeast extract (THY), and brain–heart infusion broth (BHI), though a significant increase in LukGH-mediated cytotoxicity was observed during growth in LB (Figure 3A, Figure 3B, Figure 3C, and Figure 3D, respectively). Taken together, these findings show that PVL is the dominant extracellular cytotoxic factor causing PMN lysis that is produced by USA300.

### 3.3. Lysis of Human PMNs Following Phagocytosis of USA300 Is Primarily Mediated by Bicomponent Leukocidins

**Figure 4:**
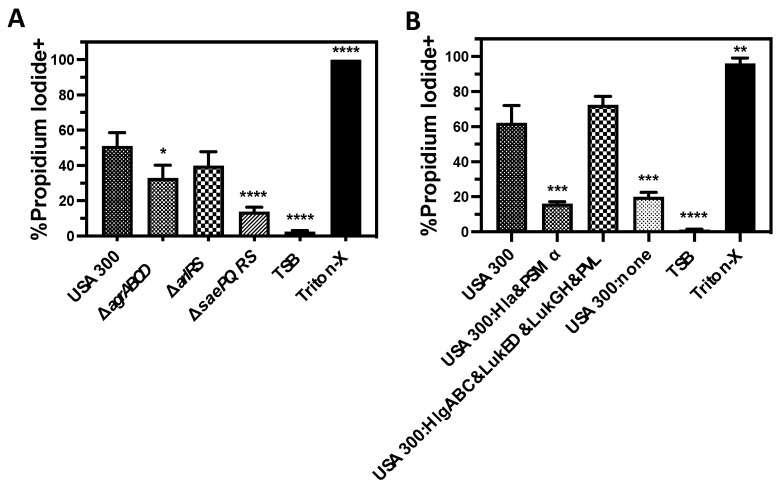
Human PMN plasma membrane permeability following phagocytosis of USA300 is primarily mediated by the bicomponent leukocidins. Flow cytometry was used to assess the percentage of purified human PMNs permeable to propidium iodide after phagocytosis of (**A**) USA300, a deletion mutant of the AgrABCD two-component system in USA300 (USA300Δ*agrABCD*), a deletion mutant of the ArlRS two-component system in USA300 (USA300Δ*arlRS*), or a deletion mutant of the SaePQRS two-component system in USA300 (USA300Δ*saePQRS*) as well as (**B**) USA300, a deletion mutant of the bicomponent leukocidins *hlgABC*, *lukED, lukGH*, and *pvl* in USA300 that still expresses Hla and PSMα (USA300:Hla&PSMα), a deletion mutant of the pore-forming toxins *hla* and *psm*α that still expresses the bicomponent leukocidins (USA300:HlgABC&LukED&LukGH&PVL), or a deletion mutant of *hlgABC*, *lukED*, *lukGH*, *pvl*, *hla*, and *psm*α in USA300 (USA300:none). For both panels, PMNs were analyzed 90 min after phagocytosis and included cells treated with tryptic soy broth (TSB) alone or TSB with 0.5% Triton X-100 (Triton-X) as controls. All data are the mean ± SEM of at least 3 independent experiments with * *p* ≤ 0.05, ** *p* ≤ 0.01, *** *p* ≤ 0.001, and **** *p* ≤ 0.0001 relative to USA300 as determined by repeated measures one-way ANOVA with Tukey’s multiple comparison test.

To further examine the relative impact of different cytotoxins produced by *S. aureus* on PMN viability, we measured PMN plasma membrane permeability following phagocytosis of live USA300 deletion mutants (Figure 4). Parallel to cytotoxicity assays using extracellular factors produced by USA300, we found that loss of the SaePQRS two-component system caused a major decrease in human PMN plasma membrane permeability following phagocytosis of USA300 as previously reported [56,57] (Figure 4A). Deletion of the AgrABC or ArlRS two-component systems also decreased plasma membrane permeability following phagocytosis as previously shown by others [47,68] but to a lesser degree than loss of SaePQRS. As with the cytotoxicity of extracellular proteins produced by USA300, the bicomponent leukocidins were essential for compromising PMN plasma membrane integrity following phagocytosis with no observed contribution from Hla or PSMα (Figure 4B). 

### 3.4. LukGH Is the Primary Initial Cause of Human PMN Destruction Following Phagocytosis of USA300

**Figure 5:**
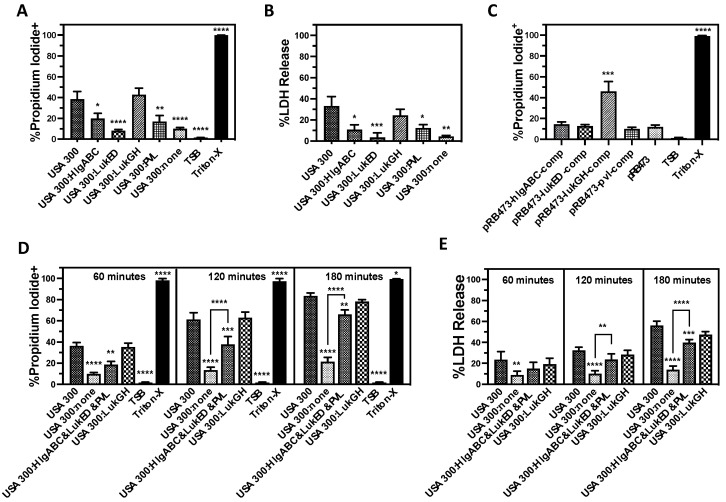
LukGH is the major factor causing human PMN lysis following phagocytosis of USA300. Phagocytosis by purified human PMNs of USA300, different USA300 deletion mutants of bicomponent leukocidins in USA300 that express only HlgAB and HlgCB (USA300:HlgABC), LukED (USA300:LukED), LukGH (USA300:LukGH), or PVL (USA300:PVL), as well as a USA300 deletion mutant of all the bicomponent leukocidins (USA300:none) for 120 min followed by quantification of (**A**) plasma membrane permeability to propidium iodide and (**B**) lactate dehydrogenase (LDH) release. (**C**) USA300 lacking all of the bicomponent leukocidins was transformed with the pRB473 control or pRB473 encoding *hlgABC*, *lukED*, *lukGH*, or *pvl* and examined for the ability to cause human PMN plasma membrane permeability following phagocytosis by human PMNs. Phagocytosis assays using purified human PMNs and USA300, a deletion mutant of all the bicomponent leukocidins in USA300 (USA300:none), a deletion mutant of LukGH that still expresses the other bicomponent leukocidins (USA300:HlgABC&LukED&PVL), or a deletion mutant of all the bicomponent leukocidins except for LukGH (USA300:LukGH) that were assessed at 60, 120, or 180 min for (**D**) plasma membrane permeability to propidium iodide and (**E**) lactate dehydrogenase (LDH) release. For all panels, PMNs were treated with tryptic soy broth (TSB) alone or TSB with 0.5% Triton X-100 (Triton-X) as controls. All data are the mean ± SEM of at least 4 independent experiments with * *p* ≤ 0.05, ** *p* ≤ 0.01, *** *p* ≤ 0.001, and **** *p* ≤ 0.0001 relative to USA300 (Panels (**A**,**B**,**D**,**E**) or pRB473 (panel (**C**)) as determined by repeated measures one-way ANOVA with Tukey’s multiple comparison test.

To elucidate the relative importance of individual bicomponent leukocidins on PMN lysis following phagocytosis of *S. aureus*, we examined the cytotoxicity of USA300 deletion mutants that express only one of these leukocidins using human PMN phagocytosis assays (Figure 5). In contrast with previous assays showing that PVL is the primary extracellular cytotoxic factor produced by USA300 against human PMNs, we found that LukGH was the most important cytotoxin causing initial human PMN plasma membrane permeability and LDH release following phagocytosis of USA300 (Figure 5A,B). The expression of PVL alone or HlgAB and HlgCB alone also increased PMN lysis, though this change was not significant. However, the combined expression of PVL, HlgAB, HlgCB, and LukED significantly increased PMN destruction 120 min following phagocytosis of USA300 and caused cytotoxicity nearing LukGH expression alone by 180 min (Figure 5D,E). These results were further supported by complementation of USA300 lacking all the bicomponent leukocidins with a plasmid encoding *lukGH* that rescued PMN lysis following phagocytosis, while plasmids reintroducing the other bicomponent leukocidins had no observable impact (Figure 5C). Collectively, these results demonstrate that LukGH is the most important cytotoxic factor causing human PMN lysis immediately following phagocytosis. 

## 4. Discussion

A large body of research has demonstrated the cytotoxicity of numerous pore-forming toxins produced by *S. aureus* against human PMNs [17,18,19,20,21,22,23,24,25,26,27,28,29,30,31,32,33,34,35,36,37,38,39,40,41,42,43,44,45,46,47,48]. However, a direct and comprehensive analysis comparing their relative contribution to PMN lysis using relevant concentrations naturally produced by *S. aureus* has been lacking. In this study, we used a library of pore-forming toxin deletion mutants in the clinically relevant MRSA strain USA300 to examine the relative cytotoxicity of each using two different PMN intoxication assays; the first measures the cytotoxicity of extracellular proteins produced by *S. aureus,* while the second examines lysis following phagocytosis of live bacteria. We found that PVL is the dominant extracellular cytotoxin causing PMN lysis produced by USA300, while initial PMN lysis following phagocytosis of USA300 is driven primarily by LukGH. 

Although PVL was the first bicomponent leukocidin to be purified and characterized [69,70], this cytotoxin is encoded in the genome of only 36% of clinical *S. aureus* isolates in the United States [71]. Notably, these PVL-positive strains include exceptionally virulent community-associated MRSA such as USA300 that have emerged within the last several decades as a prominent cause of skin and soft tissue infections [72]. While strong species specificity of this toxin has limited the usefulness of some animal models of infection to examine its importance [32,33,37,73], numerous studies have demonstrated that PVL is a potent cytotoxin against human PMNs [31,32,33,36,37,38]. The results in this study found that PVL was the dominant cytotoxic factor against human PMNs produced by USA300 under all the growth conditions tested. 

We found the other pore-forming toxins had a relatively minor contribution to human PMN lysis caused by extracellular components expressed by USA300 as compared to PVL. We suspect that these virulence factors are also important but play dominant roles during other stages of pathogenesis that require specific experimental approaches to highlight their relevance. This is illustrated by the minimal cytotoxicity induced by LukGH during intoxication assays using extracellular components, in contrast to the prominent impact of this bicomponent leukocidin on PMN destruction following phagocytosis as discussed below. We propose that alternate experimental approaches will also demonstrate the context-dependent importance of the other pore-forming toxins for USA300 virulence. For example, it has been shown that *hlgABC* is highly upregulated by USA300 immediately following exposure to human blood [27], but the translation of HlgC in this strain is strongly reduced relative to HlgB due to a single point mutation in the 5′ untranslated region of the *hlgCB* operon [74]. This indicates that stimuli associated with human blood triggers HlgAB-mediated cytotoxicity to enhance bacterial survival, and assays that include this trigger and examine cell types susceptible to HlgAB may be needed to elucidate a dominant role for this bicomponent leukocidin in USA300 pathogenesis.

As opposed to the majority of pore-forming toxins expressed by *S. aureus*, LukGH was only recently identified by genomic sequencing [11]. This is somewhat surprising given that this bicomponent leukocidin is encoded in the genome of almost all *S. aureus* strains [15]. The sequence of LukGH is only 30% homologous with other bicomponent leukocidins [11], suggesting it has functions that are distinct from other cytotoxins. The first published study characterizing LukGH by Ventura et al. [45] demonstrated it is one of the most abundantly expressed proteins on the surface of USA300 and plays a significant role in causing human PMN lysis following phagocytosis, findings supported by subsequent research [41,42,75]. This led some to speculate that this bicomponent leukocidin plays a primary role during initial contact of *S. aureus* with host cells [12]. It has also been shown that unlike the other *S. aureus* pore-forming toxins, LukGH is pre-assembled in dimers prior to engagement with the host cell membrane [29,75,76]. Our findings demonstrate that LukGH is the primary factor causing human PMN lysis immediately following phagocytosis of USA300, with a significant contribution from the combined influence of all other USA300 cytotoxins only observed at later times. Based on these previously published reports and results from this study, we hypothesize that LukGH is poised on the bacterial surface in pre-assembled active form to immediately compromise the plasma membrane of human PMNs upon initial contact with *S. aureus*. 

The importance of PVL for extracellular cytotoxicity against human PMNs and LukGH for causing PMN destruction following phagocytosis suggests they each play significant roles during different aspects of *S. aureus* disease. For example, the concentration of extracellular cytotoxins produced by *S. aureus* would be minimal immediately following inoculation into human tissue, yet survival following initial engagement with phagocytes is critical for subsequent pathogenesis. Under these conditions, expression of LukGH would give *S. aureus* a significant advantage in surviving phagocytosis and disseminating into host tissue to initiate disease. In contrast, the expression of high concentrations of extracellular cytotoxins by *S. aureus* during human infection will occur in an established abscess where PMNs and other immune cells have surrounded and isolated concentrated *S. aureus* [77]. High levels of PVL are likely produced by USA300 under these conditions where it would play an important role destroying incoming human PMNs. Indeed, expression of PVL is strongly correlated with human skin and soft tissue infections generally characterized by abscessed *S. aureus* [72,78]. 

This research profiles the relative susceptibility of human PMNs to *S. aureus* pore-forming toxins produced extracellularly and following phagocytosis. Taken together, our results show that PVL is the primary extracellular cytotoxic factor compromising PMN cell membrane integrity produced by USA300. In contrast, LukGH was the major cause of PMN destruction immediately following phagocytosis of USA300. These findings demonstrate that what appears to be redundant pore-forming toxins in the *S. aureus* arsenal actually play very different parts in promoting pathogenesis. 

## Figures and Tables

**Table 1:** USA300 isolates used in this study.

Strain	Genes Deleted; Complemented	Toxin Genes Present (↓ = Downregulated)
USA300 strain LAC	none	*hlgABC*, *lukED*, *lukGH*, *pvl*, *hla*, and *psmα* [54]
USA300Δ*agrABCD*	*agrABCD*	*↓hlgABC*, *↓lukED*, ↓*lukGH*, ↓*pvl*, ↓*hla*, and *↓psm* [11,13,16]
USA300Δ*saePQRS*	*saePQRS*	*↓hlgABC*, *↓lukED*, ↓*lukGH*, ↓*pvl*, ↓*hla*, and *psmα* [13,59]
USA300Δ*arlRS*	*arlRS*	*↓hlgABC*, *lukED*, ↓*lukGH*, ↓*pvl*, *hla*, and *psmα* [62]
USA300Δ*hlgABC*	*hlgABC*	*lukED*, *lukGH*, *pvl*, *hla*, and *psmα*
USA300Δ*lukGH*	*lukGH*	*hlgABC*, *lukED*, *pvl*, *hla*, and *psmα*
USA300Δ*lukED*	*lukED*	*hlgABC*, *lukGH*, *pvl*, *hla*, and *psmα*
USA300Δ*pvl*	*pvl*	*hlgABC*, *lukED*, *lukGH*, *hla*, and *psmα*
USA300Δ*psm-a*Δ*hla*	*psm-a* and *hla*	*hlgABC*, *lukED*, *lukGH*, and *pvl*
USA300Δ*hlgABC*Δ*lukGH*Δ*pvl*	*hlgABC*, *lukGH*, and *PVL*	*lukED, hla*, and *psmα*
USA300Δ*hlgABC*Δ*lukGH*Δ*lukED*	*hlgABC*, *lukGH*, and *lukED*	*pvl*, *hla*, and *psmα*
USA300Δ*pvl*Δ*lukGH*Δ*lukED*	*pvl*, *lukGH*, and *lukED*	*hlgABC*, *hla*, and *psmα*
USA300Δ*pvl*Δ*hlgABC*Δ*lukED*	*pvl*, *hlgABC*, and *lukED*	*lukGH*, *hla*, and *psmα*
USA300Δ*hlgABC*Δ*lukGH*Δ*pvl*Δ*lukED*	*hlgABC*, *lukGH*, *PVL*, and *lukED*	*hla* and *psmα*
USA300Δ*hlgABC*Δ*lukGH*Δ*pvl*Δ*lukED*Δ*hla*Δ*psm*	*hlgABC*, *lukAB*, *pvl*, *lukED*, *hla* and *psm-a*	none
USA300Δ*hlgABC*Δ*lukGH*Δ*pvl*Δ*lukED* pRB473-*pvl*-comp	*hlgABC*, *lukGH*, *pvl*, *lukED*; *pvl* complemented	*pvl*, *hla*, and *psmα*
USA300Δ*hlgABC*Δ*lukGH*Δ*pvl*Δ*lukED* pRB473-*hlgABC*-comp	*hlgABC, lukGH, pvl, lukED*; *hlgABC* complemented	*hlgABC, hla*, and *psmα*
USA300Δ*hlgABC*Δ*lukGH*Δ*pvl*Δ*lukED* pRB473-*lukED*-comp	*hlgABC, lukGH, pvl, lukED*; *lukED* complemented	*lukED, hla*, and *psmα*
USA300Δ*hlgABC*Δ*lukAB*Δ*pvl*Δ*lukED* pRB473-*lukGH*-comp	*hlgABC, lukGH, pvl, lukED; lukGH* complemented	*lukGH*, *hla*, and *psmα*

**Table 2:** Primers used in this study.

Primer	Sequence
agrABCD-Top_fwd	5′ - GGG GAC AAG TTT GTA CAA AAA AGC AGG CGA AGC GCC CGA AAT AAT ATT TAA CAC - 3′
agrABCD-SphI-Top_rvs	5′ - GGT GGT GCA TGC CTC CTC ACT GTC ATT ATA CGA TTT AG - 3′
agrABCD-SphI-Bot_fwd	5′ - GGT GGT GCA TGC GTC AGT TAA CGG CGT ATT CAA TTG - 3′
agrABCD-Bot_rvs	5′ - GGG GAC CAC TTT GTA CAA GAA AGC TGG GTG TAA GCC CTC TGC TGA TAT G - 3′
SaePQRS-Top_Fwd	5′ - GGG GAC AAG TTT GTA CAA AAA AGC AGG CGA AGG GGA AGT CAT TAC ACA AAC - 3′
SaePQRS-SphI-Top_Rvs	5′ - GGT GGT GCA TGC CTC CCA TTA ATG AGG GCT TC - 3′
saePQRS-SphI-Bot_fwd	5′ - GGT GGT GCA TGC CTC GGA GAG ATT GCA ATT GG - 3′
saePQRS-Bot_Rvs	5′ - GGG GAC AAG TTT GTA CAA AAA AGC AGG CGT CAT ATG GCC GTT AAA CCA CA - 3′
arlRS-SalI-Top_fwd	5′ - TGT CGA CCT CAT ATT ACG ACT TTT TC - 3′
arlRS-PstI-Top_rvs	5′ - CTG CAG TAA ACC TAA AGT GTC GTA AG - 3′
arlRS-SacI-Bot_fwd	5′ - TCA CTA TTG AGC TCT TTG TTA AAG TAG - 3′
arlRS-BamHI-Bot_rvs	5′ - AAA TGG ATC CTA TCA TAA AAT TAG TCG AAG - 3′
hlgABC-SphI-Top_Fwd	5′ - GGG GAC AAG TTT GTA CAA AAA AGC AGG CGT TCG TCA TGA TGA GCG TG - 3′
hlgABC-SphI-Top_rvs	5′ - GGT GGT GCA TGC GGT CGC AGG CGT TTA TAT AG - 3′
hlgABC-SphI-Bot_Fwd	5′ - GGT GGT GCA TGC GTG ACG ACC GTG - 3′
hlgABC-SphI-Bot_rvs	5′ - GGG GAC CAC TTT GTA CAA GAA AGC TGG GTG CGC TAA ATC AAG GGA TG - 3′
lukGH-SphI-Top_fwd	5′ - GGG GAC AAG TTT GTA CAA AAA AGC AGG CCA ATC AGG GTG GGA CAA AAC - 3′
lukGH-SphI-Top_rvs	5′ - GGG GGT GGT GCA TGC GAC GTG CAG TGT ATG AAT CTT G - 3′
lukGH-SphI-Bot_fwd	5′ - GGT GGT GCA TGC GAT TGA TAT TTG TTG ATA TGT ATC GAC ATG TG - 3′
lukGH-SphI-Bot_rvs	5′ - GGG GAC CAC TTT GTA CAA GAA AGC TGG GTC AAT GAT TTG AAC ATA GGC GCA AC - 3′
lukED-SphI-Top_fwd	5′ - GGG GAC AAG TTT GTA CAA AAA AGC AGG CGA AGT TAA GGC CTA CTT CAA TTG TC - 3′
lukED-SphI-Top_rvs	5′ - GGT GGT GCA TGC GAA ACT AAT CCT GGA GTA TAA CTG TTA G - 3′
lukED-SphI-Bot_fwd	5′ - GGT GGT GCA TGC CTA CTG ACA AAG TTG CAG CTA AC - 3′
lukED-SphI-Bot_rvs	5′ - GGG GAC CAC TTT GTA CAA GAA AGC TGG GTG TGC TCG TCG TCA AGA C - 3′
PVL-SphI-Top_fwd	5′ - GGG GAC AAG TTT GTA CAA AAA AGC AGG CCT CAT ATC ATC GCC TTT GTC C - 3′
PVL-SphI-Top_rvs	5′ - GGT GGT GCA TGC GGA ATC AAC TTC ACT GGA TAG G - 3′
PVL-SphI-Bot_fwd	5′ - GGT GGT GCA TGC CTA ACG ACA ATG TTG CAG CTA ATA G - 3′
PVL-SphI-Bot_rvs	5′ - GGG GAC CAC TTT GTA CAA GAA AGC TGG GTG AGA AAG CGC AAG TGG TG - 3′
PSMa-Top_fwd	5’′ - GGG GAC AAG TTT GTA CAA AAA AGC AGG CGT CGT CTA CCT TTC CAT GC - 3′
PSMa-SphI-Top_rvs	5′ - GGT GGT GCA TGC CTC AGG CCA CTA TAC CAA TAG - 3′
PSMa-SphI-Bot_fwd	5′ - GGT GGT GCA TGC CAG CGA TGA TAC CCA TTA AGA TTA CC - 3′
PSMa-Bot_rvs	5′ - GGG GAC CAC TTT GTA CAA GAA AGC TGG GTC GAA TGC AAG CCA ACC AC - 3′
hla-Top_fwd	5′ - GGG GAC AAG TTT GTA CAA AAA AGC AGG CGA AGT CCA TAC AAA ATC CGC ATC - 3′
hla-BamHI-Top_rvs	5′ - GGT GGT GGA TCC CTA TCT ACT TGA TTT GCT TTC CTG AC - 3′
hla-BamHI-Bot_fwd	5′ - GGT GGT GGA TCC CAA TTT CGA GGG TTA GTC AAA GTT G - 3′
hla-Bot_rvs	5′ - GGG GAC CAC TTT GTA CAA GAA AGC TGG GTG CAA TAC TTT ATT GTC CCA TGA TTA GTG - 3′
pvl-EcoRI-comp_fwd	5′ - AGG AGG GAA TTC GTT TGG TAA TGA ACG GGT TTT TTT CG - 3′
pvl-BamHI-comp_rvs	5′ - GGT GGT GGA TCC CAA TTA AGA CGT GGT TAC CCT AAT ATA G - 3′
hlgABC-SacI-comp_fwd	5′ - GGT GGT GAG CTC CAG TTA ATT CGA AAA CGC TTA CAA ATG G - 3′
hlgABC-BamHI-comp_rvs	5′ - GGT GGT GGA TCC CTG TTG GCG ACC GTG - 3′
lukED-SacI-Comp_fwd	5′ - GGT GGT GAG CTC CCA TGA GAG TAG AAG CTT CAG - 3′
lukED-BamHI-Comp_rvs	5′ - GGT GGT GGA TCC GAA GTT AAG ACC CAC TTC AAT TGT C - 3′
lukGH-EcoRI-comp_fwd	5′ - GGT GGT GAA TTC GTA TCA ACG ATC TTA TTA ACG CTG - 3′
lukGH-BamHI-comp_rvs	5′ - GGT GGT GGA TCC CTA CAT TCT ATG TAG CAG GCA AC - 3′

## Data Availability

The raw data supporting the conclusions of this article will be made available by the authors on request.
